# Different NaCl-Induced Calcium Signatures in the *Arabidopsis thaliana* Ecotypes Col-0 and C24

**DOI:** 10.1371/journal.pone.0117564

**Published:** 2015-02-27

**Authors:** Sandra M. Schmöckel, Alexandre F. Garcia, Bettina Berger, Mark Tester, Alex A. R. Webb, Stuart J. Roy

**Affiliations:** 1 Australian Centre for Plant Functional Genomics and The University of Adelaide, Adelaide, Australia; 2 Centre for Desert Agriculture, Division of Biological and Environmental Sciences and Engineering, King Abdullah University of Science and Technology, Thuwal Saudi Arabia; 3 The Plant Accelerator, Australian Plant Phenomics Facility and The University of Adelaide, Adelaide, Australia; 4 Department of Plant Sciences, The University of Cambridge, Cambridge, United Kingdom; University of Delhi South Campus, INDIA

## Abstract

A common feature of stress signalling pathways are alterations in the concentration of cytosolic free calcium ([Ca^2+^]_cyt_), which allow the specific and rapid transmission of stress signals through a plant after exposure to a stress, such as salinity. Here, we used an aequorin based bioluminescence assay to compare the NaCl-induced changes in [Ca^2+^]_cyt_ of the Arabidopsis ecotypes Col-0 and C24. We show that C24 lacks the NaCl specific component of the [Ca^2+^]_cyt_ signature compared to Col-0. This phenotypic variation could be exploited as a screening methodology for the identification of yet unknown components in the early stages of the salt signalling pathway.

## Introduction

Worldwide, approximately 20% of the irrigated agricultural land is affected by soil salinity [1]. This leads to a significant decrease in crop yield, ultimately impacting revenue and food security. In many crop species salinity stress is predominantly due to high levels of sodium chloride (NaCl) in the soil. NaCl will hereby be referred to as salt.

Plant salinity stress can be categorised into two phases, the initial shoot ion-accumulation independent stress (sometimes called “osmotic” stress) and the later shoot ion-dependent stress (ionic stress) [2,3]. The shoot ion-accumulation independent stress occurs as soon as the plant encounters salt in the soil and results in an immediate reduction in the shoot growth rate [4]. Ionic stress is caused by the accumulation of ions such as Na^+^ and Cl^-^ in the cytosol of cells in the shoot, resulting in the inhibition of cellular processes and induces premature leaf senescence [2]. Plant responses to the ion independent component of salt stress occur in the shoot prior to the accumulation of toxic concentrations of ions in cells. This suggests that a signalling pathway exists whereby perception of salt at the root/soil interface is communicated to the shoot, before the onset of ionic stress.

Currently, the transmission of NaCl induced stress signals in plants is only partially understood. In both animal and plant signalling pathways, calcium ions (Ca^2+^) play an important role as second messengers in the cytosol, mediating the response to developmental and environmental stimuli. Alterations in the cytosolic free Ca^2+^ concentration ([Ca^2+^]_cyt_) are involved in a variety of plant signalling pathways such as abiotic stress responses [5,6], control of stomatal aperture [7,8] and interactions with pathogenic and symbiotic microorganisms [9,10]. It has been hypothesised that alterations in [Ca^2+^]_cyt_ follow a spatial and temporal pattern (referred to as a calcium signature), inducing a stimulus-specific response [5,11,12].

Changes in [Ca^2+^]_cyt_ have also been linked to the salt stress signalling pathway. Immediate increases of [Ca^2+^]_cyt_ can be observed when a plant is challenged with NaCl [13–16]. The NaCl-induced increases in [Ca^2+^]_cyt_ can be oscillatory [13, 14] with evidence of cell- and stimulus-type encoding in the NaCl-induced [Ca^2+^]_cyt_ signals in the leaves [17]. In *Arabidopsis thaliana* several components of a signalling pathway for salt stress have been identified by the characterisation of *salt overly sensitive* (*sos*) mutants [18,19]. NaCl-initiated increases in [Ca^2+^]_cyt_ are sensed by Salt Overly Sensitive 3 [SOS3; calcineurin B-like protein 4 (AtCBL4)] and the binding of Ca^2+^ facilitates the interaction of SOS3 with SOS2 (CBL-interacting protein kinase 24, AtCIPK24) [19,20]. This complex has been shown to activate the Na^+^/H^+^-antiporter SOS1 by phosphorylation, resulting in the transport of Na^+^ out of the cell and the reduction of [Na^+^]_cyt_ [21,22]. Early speculations arose that SOS1 could possibly act as a sensor for NaCl due to the protein’s long C-terminus and other characteristics [23], however no evidence supporting this hypothesis has been put forward.

It appears that many rapidly responding genes are common to different abiotic stress treatments, while genes that are expressed differently at later time points appear stress specific [24]. Interestingly, a study examining the expression profiles of salt responsive genes in different ecotypes of Arabidopsis identified the ecotype C24 as being less responsive to salt stress when compared to Col-0 [25]. A possible explanation for this finding is that the ecotype C24 has an alternate or defective Ca^2+^ signalling pathway [25]. Given that changes in expression levels of salt responsive genes are downstream of the salt signalling pathway, how salt stress is initially perceived by plant cells and the initiation of intracellular signalling pathways, require further investigation. As Ca^2+^ is known to play a key role in the early stages of the salt stress response, one possibility is to investigate the stress activated calcium signalling pathway, which generates stress specific calcium signatures,.

Calcium signatures can be analysed using the aequorin bioluminescence reporter system. *Apo-aequorin* encodes for a precursor protein which forms functional aequorin when supplemented with the prosthetic group, coelenterazine [26]. In this system, Ca^2+^ binds to aequorin, leading to the emission of photons that can be measured using a luminometer, thereby giving an indication of total [Ca^2+^]_cyt_ present at any given time. It has been used previously to measure increases in [Ca^2+^]_cyt_ to analyse the response of Arabidopsis seedlings to abiotic stresses such as drought, cold and salt stress [13–15,17,27].

Here we show that the analysis of calcium signatures in response to salt stress might offer an opportunity to further investigate components of the salt signalling pathway. We provide evidence that calcium signatures evoked by salt treatment vary between the responsive ecotype Col-0 and the less-responsive ecotype C24. In addition, the importance of the temporal aspect of alterations in [Ca^2+^]_cyt_ will be discussed.

## Material and Methods

### Plant material and growth conditions

Luminometric experiments were performed using previously developed *Arabidopsis thaliana* ecotypes Col-0 and C24 expressing *APOAEQUORIN* under control of the *CaMV-35S* promoter [15,28]. Seeds were surface sterilised with 70% (v/v) ethanol for 5 min, washed with sterile water five times and sown onto petri dishes containing ½ strength Murashige and Skoog (Duchefa, Harlem, Netherlands) supplemented with 0.8% (w/v) Bacto agar (Becton, Dickson and Company, Sparks, MD, USA) (adjusted to pH 5.7 with KOH). The plates were incubated horizontally in a growth chamber under 12/12 h light/dark regime, at 20°C and 80 μM m^-2^ s^-1^ light intensity.

### Measurement of [Ca^2+^]_cyt_

Seedlings were grown for 13 d to 15 d before developmentally similar seedlings were placed into luminometer cuvettes (51 mm high x 12 mm diameter, Sarstedt, Leicester, UK) containing 300 μL reconstitution solution [1.4 mM CaCl_2_, 20 mM KCl, 5 mM 2-(N-morpholino)-ethansulfonic acid (MES) and 10 μM coelenterazine (Prolume, Pinetop, AZ, USA), adjusted to pH 5.5 with KOH]. The experiments were conducted in this depolarising base solution because of its simple composition as described previously [14] and its ability to impose a membrane potential similar to that of MS growth solution, -40 mV for depolarising base solution and -55 mV for MS [29]. Treatment solutions consisted of base solution (1.4 mM CaCl_2_, 20 mM KCl, 5 mM MES) supplemented with the nominated amount of NaCl or sorbitol. Ca^2+^ activity in NaCl containing treatment solutions was maintained by addition of CaCl_2_ as determined using the programme Visual MINTEQ (version 3.0, http://www2.lwr.kth.se/English/OurSoftware/Vminteq/). The osmolarity of solutions was measured using a Wescor 5520 Vapour Pressure Osmometer (Logan, UT, USA) following the manufacturer’s instructions. The osmolarity of sorbitol solutions was adjusted to the osmolarity of NaCl solutions. The luminometry and conversion of photon counts into [Ca^2+^]_cyt_ was performed exactly as has been described elsewhere [30].

### Data analysis

In order to determine whether there was a significant difference in the response curves between Col-0 and C24 the following data analysis was performed.

1. The response curves of three biological replicates for each ecotype and each treatment concentration was averaged.
2. The response curve was split into two temporal regions to separately identify two local peaks in [Ca^2+^]_cyt_. The first peak was defined as the local maximum that occurred within the first 30 seconds after stimulus onset, while the second peak was defined as the local maximum that occurred between 30 and 120 seconds after stimulus application.
3. The first and secondary peak amplitudes were determined for each ecotype and concentration from the local maximal and minimal values within the two temporal regions (1–30 s and 31–120 s).
4. The log_10_ of the second amplitude was plotted against the log_10_ of the first amplitude.
5. A linear discriminant analysis was performed using the R package atsa [31,32] to differentiate between the two ecotypes. The first and second log_10_ amplitudes were used as two-dimensional feature vectors in this analysis. A sample linear function of these two parameters (the first and second log_10_ amplitudes) was determined for each ecotype and a discriminant linear function used to differentiate between both ecotypes was obtained as the difference between the Col-0 and C24 sample linear functions [33].
6. Jackknife resampling was used to evaluate the performance of the sample discriminant functions [33], as a simple version of cross-validation. In this procedure, the discriminant linear function was derived from the training sample, removing a single observation at a time. The posterior probability that this removed sample belonged to an ecotype group was then calculated. This procedure was repeated for each member of each training sample [33].

## Results and Discussion

### NaCl induced increases in [Ca^2+^]_cyt_

**Fig 1:**
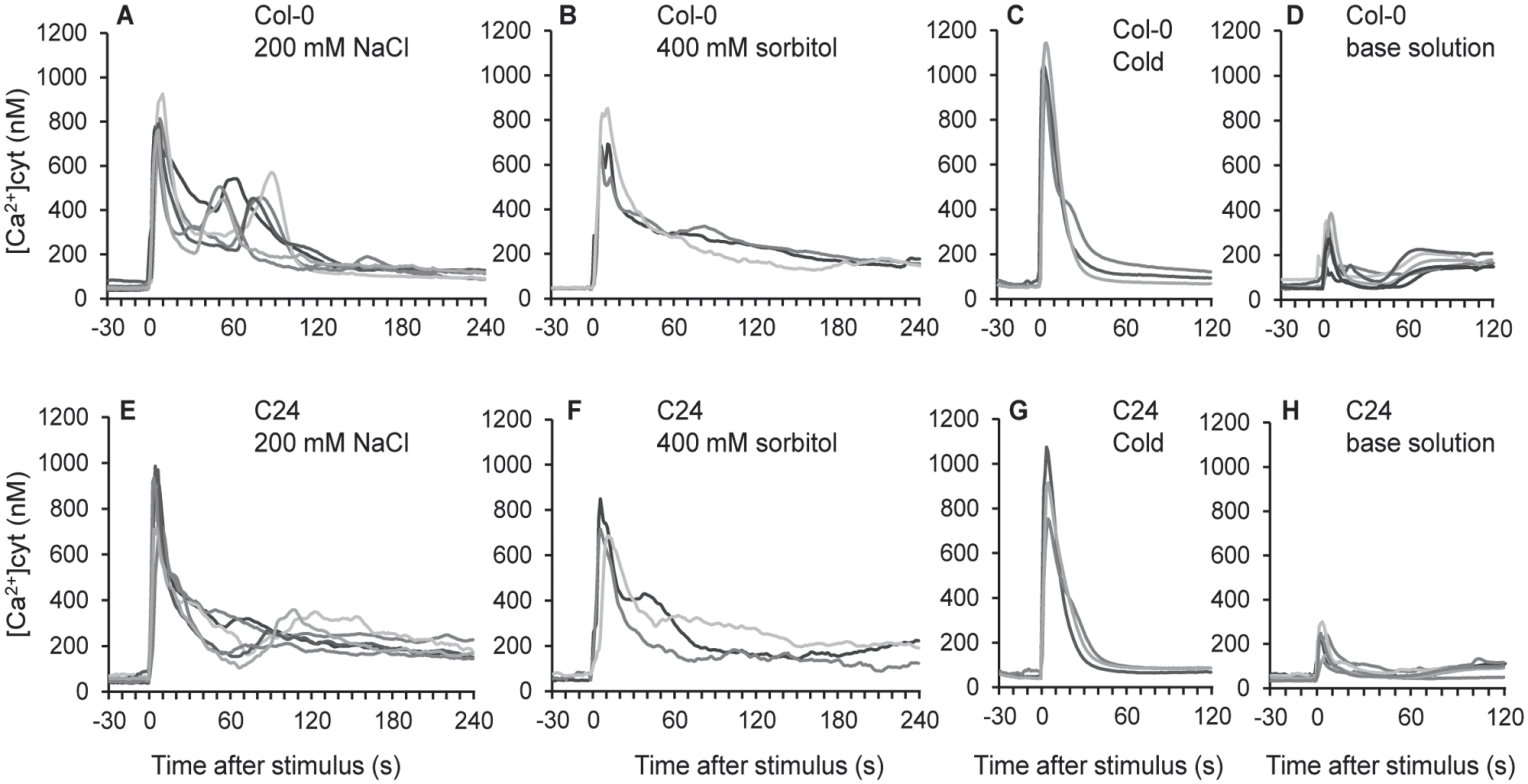
Luminometric measurements of whole Arabidopsis seedlings, constitutively expressing *aequorin*. Alterations of [Ca^2+^]_cyt_ induced by (A) 200 mM NaCl, (B) 400 mM sorbitol, (C) 9°C cold treatment or (D) base solution in ecotype Col-0 and (E) 200 mM NaCl, (F) 400 mM sorbitol, (G) 9°C cold treatment or (H) base solution in ecotype C24. Each panel contains data from three to six representative individual seedlings. Stimulus (stress) was applied at time point 0.

To investigate the early stages of the salt signalling pathway, we compared the calcium signatures of Col-0 and C24 using the aequorin bioluminescence reporter system. When Col-0 and C24 were challenged with 200 mM NaCl, 400 mM sorbitol or cold, an instantaneous increase in [Ca^2+^]_cyt_ lasting approximately 20 s of similar magnitudes for both ecotypes was observed (Fig. 1). [Ca^2+^]_cyt_ quickly declined within a few minutes to a basal level. Treatment with 200 mM NaCl resulted in a peak with a magnitude of 800 ± 39 nM and 855 ± 66 nM [Ca^2+^]_cyt_ in Col-0 and C24, respectively (Fig. 1 A and E). The same strength of osmotic stress was imposed using 400 mM sorbitol resulting in peak magnitudes of 737 ± 58 nM and 750 ± 50 nM [Ca^2+^]_cyt_ in Col-0 and C24, respectively (Fig. 1 B and F). Cold treatment was used to compare the response to a different abiotic stress; it also resulted in an instantaneous increase in [Ca^2+^]_cyt_ with magnitudes of 1052 ± 52 nM and 914 ± 93 nM [Ca^2+^]_cyt_ in Col-0 and C24, respectively (Fig. 1 C and G). The touch response invoked by application of only base solution (without other stimuli such as NaCl, sorbitol or cold) also resulted in an instantaneous, but smaller and faster, peak with a magnitude of 284 ± 37 nM [Ca^2+^]_cyt_ in Col-0 and 219 ± 27 nM [Ca^2+^]_cyt_ in C24 (Fig. 1 D and H). For all stress treatments tested, there were no significant differences in the magnitude of the first peak between ecotypes. These results are broadly in agreement with previous studies [13–15].

**Fig 2:**
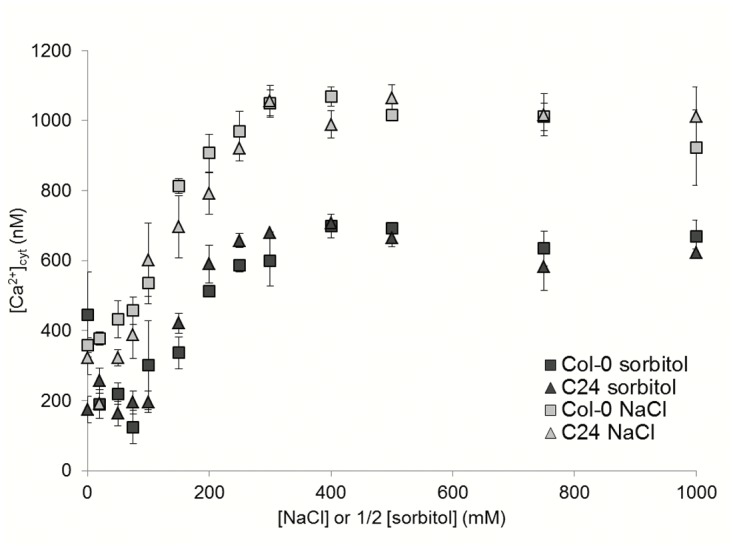
Amplitude of the first [Ca^2+^]_cyt_ peak from luminometric measurements of Col-0 and C24 in response to [NaCl] and [sorbitol] treatment. The 13 d to 15 d old Col-0 (square symbol) and C24 (triangular symbol) seedlings constitutively expressing *aequorin* were treated with a range of NaCl and corresponding equal osmotic strength of sorbitol concentrations (which can be approximated to 2 times [NaCl]). Each point represents the average peak height of the first [Ca^2+^]_cyt_ peak of three replicates, error bars indicate the standard error of the mean (S.E.M.).

The magnitudes of the initial NaCl and sorbitol induced [Ca^2+^]_cyt_ increased were dependent on stimulus strength. The amplitudes of the first peak increased with increasing concentrations of NaCl and sorbitol, but were not significantly different between ecotypes (Fig. 2). The amplitude of the first peak was determined over 13 NaCl and equivalent sorbitol concentrations in Col-0 and C24, ranging between 0 to 1 M NaCl and respective sorbitol concentrations of 0 to 2 M (Fig. 2). Over this range of concentrations, the amplitudes of sorbitol induced peaks were smaller than those of NaCl induced peaks. This suggests that the NaCl response is partially due to the osmotic and partially due to the ionic component of NaCl treatment. This is in agreement with other studies in which a small number of selected NaCl and sorbitol concentrations were tested [13–15,27]. The correlation between stimulus strength and amplitude of the first [Ca^2+^]_cyt_ peak indicates that a touch-induced increase in [Ca^2+^]_cyt_ is not the major contributor to [Ca^2+^]_cyt_ increases in this study.

### Col-0 and C24 have different calcium signatures in response to NaCl

There was a pronounced difference in the dynamics of the NaCl-induced [Ca^2+^]_cyt_ alterations between Col-0 and C24 (Fig. 1 A and E). In Col-0, the immediate elevation in [Ca^2+^]_cyt_ to 800 ± 39 nM was followed by a second increase to 656 ± 60 nM [Ca^2+^]_cyt_ which occurred between 30–120 seconds after NaCl application, and lasted approximately 30 s (Fig. 1 A). In C24, the first peak to 855±66 nM [Ca^2+^]_cyt_ was not followed by a distinguishable second increase (Fig. 1 E). The calcium signatures for salt treated Col-0 seedlings were biphasic—the signatures in C24 only contained the initial peak. Notably, the occurrence of the secondary rise in [Ca^2+^]_cyt_ in Col-0 differed in individual seedlings (Fig. 1 A).

**Fig 3:**
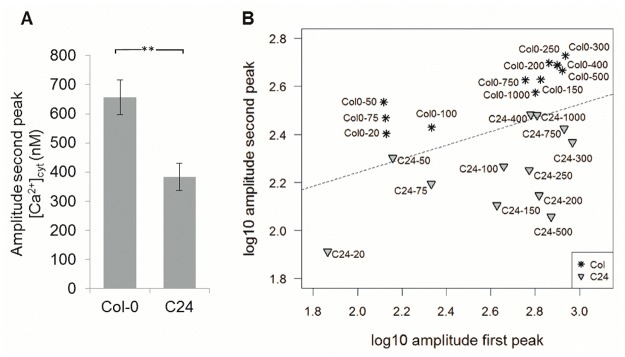
Analysis of amplitudes of [Ca^2+^]_cyt_ peaks in response to [NaCl] treatment. (A) Amplitude of the second [Ca^2+^]_cyt_ peak of Col-0 and C24 seedlings constitutively expressing *aequorin* treated with 200 mM NaCl. (B) Classification of Col-0 and C24 ecotypes based on linear discriminant analysis of amplitudes of the first and second [Ca^2+^]_cyt_ peak of Col-0 and C24 seedlings stimulated with a range of NaCl concentrations. Each point represents the average height of [Ca^2+^]_cyt_ peaks from three replicates. Error bars indicate the standard error of the mean (S.E.M.). Asterisk in (A) indicates the significance based on a t-test, with ** indicating p<0.01.

Differences in the secondary rises in [Ca^2+^]_cyt_ between Col-0 and C24 were compared, assessing the bimodal characteristics. The amplitude of the second peak was significantly different between the ecotypes Col-0 and C24 when challenged with 200 mM NaCl, with values of 656 ± 60 nM [Ca^2+^]_cyt_ and 383 ± 46 nM [Ca^2+^]_cyt_, respectively (Fig. 3 A).

The observed differences provide evidence that the early stages of the salt signalling pathway might differ in these ecotypes. The salt signalling pathway is triggered by a NaCl stimulus and initiates a signalling cascade, which results in the influx of Ca^2+^ into the cytosol and thereby forming the calcium signature. This calcium signature is then thought to encode for the stimulus specific response. Consequently, the salt sensor, the signalling molecules, the Ca^2+^ influx system or an as yet unidentified component could be dysfunctional or absent in C24, leading to an altered calcium signature. Equally, the differences in observed calcium signatures could be due to differences in the propagation of the cytosolic calcium increases through the plant. This altered calcium signature could be the cause for the reduced responsiveness of C24 as measured by gene expression by Jha *et al*. (2010).

A range of 12 different NaCl concentrations (20 mM to 1 M) was used to further analyse the peak behaviour of NaCl induced [Ca^2+^]_cyt_ alterations in Col-0 and C24. To analyse whether it was possible to discriminate between the two ecotypes, the commonly-used linear discriminant analysis was performed. The base 10 logarithm of the amplitude of the second peak was plotted against the base 10 logarithm of the amplitude of the first peak, with the linear discriminant function drawn in the graph as a dotted line (Fig. 3 B). A clear pattern was visible, distinguishing NaCl induced [Ca^2+^]_cyt_ alterations for Col-0 and C24. Values corresponding to Col-0 were generally in the upper part of the graph and values corresponding to C24 values were generally in the lower part of the graph, showing that the amplitudes of the second peak discriminated between the ecotypes (Fig. 3 B).

Jackknifed posterior probabilities were determined to estimate the cases of misclassification of data with the Col-0 group or C24 group. The jackknifed posterior probabilities of being a Col-0 ecotype for the Col-0 group ranged from 0.912 to 0.999, for all stimuli classified as Col-0 ecotype (Table 1). The C24 probabilities for the C24 group ranged from 0.157 to 1.000. For most NaCl concentrations, the linear discriminant function could be used to discriminate between the ecotypes Col-0 and C24. The highest jackknifed posterior probabilities for Col-0 and C24 together occur at a stimulus concentration of 200 mM [NaCl], with posterior probabilities of 0.997 and 1.000 respectively. At this stimulus concentration, it is likely that the Ca^2+^ signature derived from a Col-0 plant would be associated with the Col-0 group and vice versa, the Ca^2+^ signature derived from a C24 plant would be associated with the C24 group.

**Table 1:** Jackknifed posterior probability that each individual stimulus belongs to an ecotype group defined in Fig. 2 B.

Stimulus (mM NaCl)	Col-0 jackknifed posterior probability	C24 jackknifed posterior probability
20	0.952	1.000
50	0.999	0.157
75	0.993	0.976
100	0.912	0.988
150	0.983	1.000
200	0.997	1.000
250	0.994	0.997
300	0.997	0.980
400	0.994	0.261
500	0.987	1.000
750	0.990	0.888
1000	0.943	0.340

This discrimination may allow use of this aequorin-based system for a genetic screen of a Col-0 x C24 population to identify the underlying gene causing the different Ca^2+^ signatures in response to a NaCl stimulus. It has previously been suggested by Tracy *et al*. [14] that the second peak in [Ca^2+^]_cyt_ is induced by the ionic component of NaCl, as it does not occur when the same strength of osmotic stress is applied using sorbitol. The results presented here support this hypothesis, by displaying the biphasic characteristics of NaCl induced [Ca^2+^]_cyt_ alterations and monophasic sorbitol- and cold-induced [Ca^2+^]_cyt_ alterations for the Col-0 ecotype (Fig. 1 A and B).

Previous research has often focussed on describing [Ca^2+^]_cyt_ alterations predominantly based on the amplitude of the first peak and Ca^2+^ signatures over time were often averaged [13–15,27]. Secondary peaks would have been masked by averaging the signature between samples due to the variability of the time of occurrence with peaks between samples being out of phase. The bimodal, or possibly oscillatory nature of the NaCl-induced [Ca^2+^]_cyt_ signal may be revealed by reducing the number of cells recorded [14,34] and/or by focusing on specific cell types in which NaCl-induced [Ca^2+^]_cyt_ signals are particularly oscillatory (e.g. the spongy mesophyll) [17] and root epidermis [15]. The bimodal nature may also be explained by wave-like dynamics of NaCl-induced [Ca^2+^]_cyt_ signals along the root towards the shoot, which have recently been described using local NaCl stimuli to the root tip of Arabidopsis seedlings [14,35].

Any of the three main stages in the early signalling pathway (salt sensing, signal transmission or Ca^2+^ influx) offer an explanation for the differences in [Ca^2+^]_cyt_ signatures in Col-0 and C24. However, the molecular identity of the salt sensor, signal transmission molecules and the Ca^2+^ influx systems are poorly understood and it cannot be excluded that a completely different mechanism is underlying these differences in [Ca^2+^]_cyt_ alterations. On the other hand, it is of great advantage that the genomes of Col-0 and C24 have been fully sequenced [36], making a genetic approach a useful tool to investigate the early stages of the salt signalling pathway.

A high throughput genetic screen of a Col-0 x C24 population, based on the aequorin bioluminescence system, could lead to the identification of as yet unknown components in the early stages of the salt signalling pathway.
